# A double layer oral film loaded with doxepin hydrochloride for the treatment of chemotherapy-induced oral mucositis

**DOI:** 10.3389/fphar.2025.1673190

**Published:** 2025-10-17

**Authors:** Peiyan Wang, Tianlu Wang, Hui Zhang, Pei Sun, Changqing Yuan, Yiqing Guo, Zhaochen Liu, Mengyu Jiao, Jingwen Chi, Kexin Wang, Jing Deng, Xiaofei Yu

**Affiliations:** ^1^ Department of Stomatology, The Affiliated Hospital of Qingdao University, Qingdao, China; ^2^ School of Stomatology, Qingdao University, Qingdao, China; ^3^ Dental Digital Medicine & 3D Printing Engineering Laboratory of Qingdao, Qingdao, China

**Keywords:** chemotherapy-induced oral mucositis, 3D printing, oral mucosal adhesive materials, doxepin hydrochloride, wound healing

## Abstract

Chemotherapy-induced oral mucositis is a common complication in cancer therapy. Multiple studies demonstrate that doxepin mouthwash is effective in controlling CIOM pain, while research on its ulcer healing effects and underlying mechanisms remains limited. Additionally, local adhesive formulations of doxepin for oral application are still lacking. In this study, we developed a bilayer oral patch using 3D-printing technology for the localized sustained release of doxepin hydrochloride. This patch demonstrated favorable characteristics, including thinness, flexibility, and excellent mucosal adhesion, while promoting ulcer healing in CIOM animal models. Further investigation revealed that the healing mechanism involves the alleviation of oxidative stress and attenuation of excessive inflammatory responses. The doxepin hydrochloride patch developed in this study provides a novel pharmaceutical option for clinical management of CIOM.

## 1 Introduction

Chemotherapy-induced oral mucositis (CIOM) refers to oral mucosal lesions characterized by erythema, erosion, ulceration, pseudomembrane formation, and atrophy that occur in patients with cancer during anticancer treatment, following the administration of chemotherapeutic agents, molecularly targeted drugs, or immunotherapeutic agents (Lalla et al., 2014; Zheng et al., 2025; Riley et al., 2017; Jin et al., 2025; He et al., 2025; Heidari and Salmanpour, 2023). Among patients receiving chemotherapy for head and neck cancer, the incidence of CIOM can reach as high as 59.4%–100% (Elting et al., 2007; Bahari et al., 2025).

For CIOM lesions presenting with established oral ulcers, current clinical protocols advocate pharmacological interventions to alleviate the symptoms and promote mucosal healing (Kang et al., 2024; Shao et al., 2021; Dos Santos et al., 2025; Chen et al., 2024; Parra-Rojas et al., 2025; Sun et al., 2025). Doxepin, a tricyclic antidepressant primarily indicated for depressive disorders and neurotic conditions, has demonstrated significant efficacy in pain management when administered as a topical mouthwash for CIOM, as evidenced in multiple recent clinical trials (Leenstra et al., 2014; Sio et al., 2019). Notably, the ulcer-healing properties of doxepin have been well documented in duodenal ulcer treatments (Andersen et al., 1984). However, relatively few topical adhesive dosage forms of doxepin are available, and the underlying mechanisms related to its healing effects remain unclear (Samiraninezhad et al., 2023).

The oral cavity constitutes a dynamic and moist microenvironment that poses considerable challenges for localized drug delivery (Yan et al., 2017). The current intraoral therapeutic modalities include local injection therapy, liquid-based mouth rinses, and topical pastes/adhesive patches (de Vries et al., 1991; Fini et al., 2011; Uzunoğlu et al., 2021; Liu et al., 2024; Zhang et al., 2024; Wang et al., 2024; Zhang et al., 2023). While local injections sustain therapeutic drug concentrations for longer periods, their invasive nature, coupled with significant local pain, markedly reduces patient compliance. Rinsing formulations suffer from suboptimal drug retention and unavoidable complications, such as inadvertent swallowing. Mucoadhesive patches or films represent an ideal non-invasive drug delivery system because of their user-friendly application; however, maintaining prolonged adhesion on wet mucosal surfaces remains a formidable challenge in dental biomaterials (Hu et al., 2021; Feng et al., 2024; Mohsen et al., 2021).

Inspired by marine organisms, such as mussels, whose catechol-functionalized polymers enable robust wet adhesion, researchers have developed bioadhesives, such as gelatin-dopamine (Gel-DOPA), demonstrating promising interfacial bonding (Guo et al., 2020; Hou et al., 2025). In this study, a double-layer, Gel-DOPA and doxepin hydrochloride, mucoadhesive film was engineered via 3D-printing technology. We hypothesize that the novel doxepin formulation could improves the healing of oral mucositis.

## 2 Materials and methods

### 2.1 Materials

Gelatin type A from porcine skin in powder form were provided by MKbio (Shanghai, China). Doxepin hydrochloride were obtained from MedChemExpress (Shanghai, China). Artificial saliva, hydroxypropyl methylcellulose (HPMC), N-(3-dimethylaminopropyl)-N′-ethylcarbodiimide hydrochloride (EDC) and N-hydroxysuccinimide (NHS) were purchased from Solarbio (Beijing, China). Zein powder, dopamine hydrochloride were provided by Aladdin (Shanghai, China). Isoflurane were supplied by Youcheng Bio Co. Ltd., Hong Kong. Nrf2 and Keap1 antibodies were purchased from Abclonal (United States).

### 2.2 Animals

This study received approval from the ethics committee of The Affiliated Hospital of Qingdao University (registration number: QDFYWZLL29569). The 6-week-old male Sprague-Dawley rats used in this experiment were purchased from the Jinan PengYue Experimental Animal Breeding Farm (Jinan, China). All the experimental protocols were approved by the Animal Committee of the Affiliated Hospital of Qingdao University. The rats were randomly divided into cages with five rats per cage, housed under controlled conditions with *ad libitum* access to food and water, a 12-h light/dark cycle, and maintained at a constant temperature (25 °C ± 1 °C) and relative humidity (60% ± 10%). All rats underwent a 2-week acclimatization period prior to the experiments.

### 2.3 Preparation and characterization of a wet adhesive double layer oral film loaded with doxepin hydrochloride

#### 2.3.1 Preparation and characterization of the bioadhesive Gel-DOPA

According to the method described by Gowda et al. (2020), dopamine hydrochloride was conjugated to gelatin A (from porcine skin) in the presence of EDC and NHS. Specifically, 1.0 g of gelatin-A and 250 mg of dopamine hydrochloride were dissolved in 50 mL of MES buffer. Subsequently, appropriate amounts of EDC and NHS were added to the mixture. The mixture was then shaken at 100 rpm in the dark at 37 °C for 24 h. The resulting solution was purified using a dialysis membrane against deionized water for four cycles. The final Gel-DOPA product was lyophilized and stored at −20 °C (Figure 2B) (Brennan et al., 2017; Lee et al., 2006). Approximately 1 mg of lyophilized powder sample was thoroughly mixed with 100 mg of spectroscopically pure potassium bromide powder in an agate mortar and ground homogeneously. The mixture was then compressed under a pressure of 10 MPa to form a transparent pellet for scanning. Fourier transform infrared spectroscopy (FT-IR) was performed to detect the surface functional groups in the synthesized Gel-DOPA, raw gelatin, and dopamine hydrochloride, confirming the formation of the target product. FT-IR was performed over a range of 350–7,800 cm^-1^ at a scanning resolution of 2 cm^-1^ over 32 scans using an FT-IR spectrometer (Nicolet IZ10, Thermo Fisher Scientific, United States).

#### 2.3.2 Preparation of the film backing layer

The backing-layer solution was prepared by mixing 75% ethanol, zein powder, and glycerol at a mass ratio of 250:25:1 (Yan et al., 2024). Subsequently, 7 mL of this solution was coated onto a 90-mm diameter Petri dish and vacuum-dried at 65 °C for 2 h to form a backing layer film (Figure 2A).

#### 2.3.3 Preparation of adhesion and drug-loaded layers

The adhesive and drug-loaded layers of the film were fabricated using 3D printing. The ink formulations for 3D printing are as follows: doxepin hydrochloride, 250 mg; Gel-DOPA, 200 mg; HPMC, 200 mg; CARBOPOL, 200 mg; ethanol, 4.7 mL; and water, 4.7 mL. Ethanol, water, and a stirring bar were placed in a screw-capped glass vial. Under continuous stirring, gelatin-polydopamine, CARBOPOL, HPMC, and doxepin hydrochloride were sequentially added (Takashima et al., 2022). The vials were left overnight at room temperature. Subsequently, the formed gel was loaded into a syringe with a 27G nozzle and placed in a 3D printer (INKREDIBLE, CELLINK BIO X, Sweden). The printing parameters were set to a circular area with a diameter of 90 mm, height of 1 mm, and an air pump pressure of 42 kPa. The printed gel was air-dried overnight at room temperature and a doxepin hydrochloride-loaded oral double (DL) film was fabricated (Figure 2D).

#### 2.3.4 Powder X-ray diffraction (XRD)

Powder XRD patterns of the DL film and related powder samples were analyzed. They were obtained using a MiniFlex 600 (Rigaku Co., Tokyo, Japan) by irradiating with Cu-Kα X-rays. The tube voltage and amperage were 35 kV and 25 mA, respectively. Samples were scanned from a 2θ of 5°–90°.

#### 2.3.5 Differential scanning calorimetry (DSC)

The DSC peaks of the DL film, doxepin hydrochloride, and the excipient were measured using a differential scanning calorimeter (TGA/SDTA 851e; Mettler-Toledo, Switzerland). Approximately 2 mg of the sample was placed at the bottom of the sample pan and the temperature was increased from 25 °C to 600 °C at a rate of 10 °C/min.

#### 2.3.6 Water contact angle (WCA) analysis

The WCA of the backing and drug-carrying layers were determined using a goniometer (Theta, Biolin, Sweden). Water (5 μL) was automatically dropped onto the film. The WCA was immediately and automatically measured on both sides of the droplet. All measurements were repeated at least three times for each sample and the results were averaged.

#### 2.3.7 Determination of drug content uniformity

Three batches of DL films were prepared according to the methods outlined in Sections 2.2.2, 2.2.3. Three patches were randomly selected from each batch, and a 10 × 10 mm section was excised from each patch. These samples were dissolved in 30 mL of artificial saliva, then filtered through a 0.22-μm aqueous membrane filter. The concentration of doxepin hydrochloride in the filtered solution was analyzed using liquid chromatography (LC20A, Shimadzu 145, Japan) with reference to the method described by Sanz et al. (2015) Single-point quantification was performed using doxepin hydrochloride solutions of known concentrations and the doxepin hydrochloride content in each patch was calculated.

#### 2.3.8 *In vitro* drug release

The *in vitro* release test was performed using a Franz diffusion cell. A cellulose acetate membrane (0.22 μm) was immersed in phosphate buffer (pH 6.8) for 12 h and placed on top of the receptor compartment. A DL film (1.5 × 1.5 cm) was attached to the membrane with its drug-containing layer facing the receptor compartment. The receptor compartment was filled with 9 mL artificial saliva. The system was maintained at 37 °C with a stirring rate of 100 rpm. Aliquots were collected at predetermined time intervals (10–250 min) and the concentration of doxepin hydrochloride in the dissolution medium was quantified using HPLC (as described in Section 2.3.7) using a validated standard curve (r^2^ > 0.99).

#### 2.3.9 *Ex vivo* drug permeation studies

The *ex vivo* drug permeation experiment was conducted using porcine buccal mucosa mounted on Franz diffusion cells because its histological structure closely resembles that of the human buccal mucosa (Figueiras et al., 2009). Artificial saliva (8 mL) and a magnetic stirrer bar were added to the receptor compartment. A porcine buccal mucosal tissue sample (approximately 2 × 2 cm) was clamped between the donor and receptor compartments. A 1.5 × 1.5 cm DL film was moistened and applied to the mucosal surface. The Franz diffusion cell temperature was maintained at 37 °C with a stirring speed of 100 rpm. At designated time intervals (0.5, 1, 1.5, and 2 h), the film was removed and the receptor fluid was collected. The mucosal tissue was minced and ultrasonicated in acetone for 10 min to extract the residual drug. The amount of doxepin hydrochloride in both the receptor fluid and acetone solution was quantified to determine the total amount of permeated doxepin hydrochloride.

#### 2.3.10 Measurement of film weights and thickness

We selected two additional commercially available patch-type drugs in China for the treatment of oral ulcers as references: propolis oral film (ZIZHU PHARMACEUTICAL, China) and dexamethasone acetate oral mucoadhesive patches (Shenzhen Taitai Pharmaceutical Co., Ltd., China). The weights of the film samples were measured using a precision electronic balance with an accuracy of 0.1 mg and the relative weight of three commercially available oral ulcer patches in China were calculated based on their dimensions. The thicknesses of the film samples were measured at three randomly selected points using Vernier calipers with an accuracy of 0.001 mm (n = 5).

#### 2.3.11 Patch adhesion time determination

Each type of oral patch adhered to the buccal mucosa of the anesthetized rats (n = 5) and the adhesion of the patches was visually monitored within 0–150 min. The average time required for patch detachment from the mucosa was recorded as the *in vivo* adhesion time.

Following the method described by Perioli et al. (2004), porcine buccal mucosa was first adhered to the inner wall of a beaker using an adhesive. A patch was moistened with 50 μL of phosphate-buffered saline (PBS) and pressed onto the porcine mucosa surface for 20 s. The beaker was filled with 800 mL of PBS maintained at 37 °C. After 2 min, stirring was initiated at 150 rpm and the adhesion time of the patch was monitored for over 300 min. Three samples were tested for each patch type and the average adhesion time was calculated as the *in vitro* adhesion time of the patches.

### 2.4 Evaluation of the efficacy, mechanism, and biosafety of DL film in CIOM

#### 2.4.1 Animal experimental protocol

**FIGURE 1:**
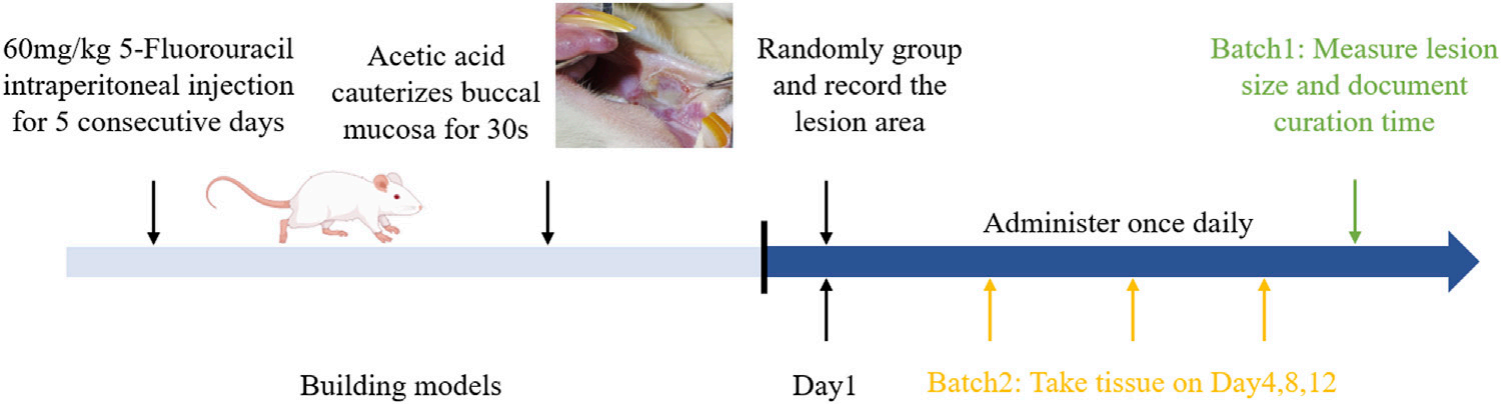
Overall animal experimental protocol.

As described in previous reports, we combined 5-FU injections with the topical application of 70% glacial acetic acid to induce chemotherapeutic oral mucosal ulcers in the buccal mucosa of rats (Çelebi et al., 2022; Lima Gde et al., 2015). Specifically, on days 1, 3, and 5, the rats received intraperitoneal injections of 0.5% 5-FU solution at a concentration of 60 mg/kg. Following the final injection on day 5, the rats were anesthetized and a small cotton ball (approximately 8-mm in diameter) soaked in 70% glacial acetic acid was applied to the buccal mucosa for 40 s to induce cauterization until the local mucosal surface appeared white. Overall experimental protocol was shown in Figure 1. In our preliminary pilot experiments, the observed effect size was f = 0.78, with a significance level (α) set at 0.05 and statistical power (1-β) set at 0.9. With a total of four groups, and using one-way ANOVA for comparisons between different groups, the calculated sample size per group was 5, resulting in a total sample size of 20 in order to evaluate the efficacy and biosafety of DL film in CIOM. To further explore the mechanism of DL film in treating CIOM, totally 35 SD rats were utilized.

#### 2.4.2 Efficacy and biosafety of DL film in CIOM

Rats were randomly divided into the following four groups: Group 1 (control group): rats that were modeled but not treated (n = 5); Group 2: modeled rats treated with doxepin patch (n = 5); Group 3: modeled rats treated with the propolis patch (n = 5); and Group 4: modeled rats treated with the dexamethasone patch (n = 5). All groups received intraperitoneal anesthesia with pentobarbital sodium administered once daily by designated experimenters at fixed time points before treatment. The modeling process was conducted in two batches. In the first batch, photographic documentation of buccal mucosal ulcer lesions was performed during treatment and ulcer area changes were analyzed using ImageJ. Macroscopic scoring was conducted on days 3, 6, and 9 of treatment using the following criteria: 1. intact oral mucosa without abnormalities; 2. moderate mucosal erythema and congestion with punctate erosions, and absence of ulcers or abscesses; 3. severe mucosal erythema and congestion with hemorrhage and small ulcers, and no abscess formation; and 4. severe mucosal erythema and congestion with hemorrhage, extensive ulceration, and abscess formation. In the second batch, the rats were euthanized after 3, 6, or 9 days of treatment. Buccal ulcer tissues were collected, fixed in neutral-buffered formalin, embedded in paraffin, and subjected to H&E staining for pathological evaluation. The major organs (heart, liver, spleen, lungs, and kidneys) were harvested after treatment for additional H&E staining to assess tissue damage and pathological alterations.

#### 2.4.3 Mechanism of DL film in CIOM

The modeling method was conducted as described in Section 2.4.1, with rats divided into three groups: Group 1, healthy rats (n = 5); Group 2, untreated modeling group (n = 15); and Group 3, doxepin-treated modeling group (n = 15). After 3, 6, and 9 days of treatment, five rats were randomly selected from Groups 2 and 3 for buccal mucosal lesion tissue collection. Part of the tissue was analyzed via RT-qPCR to detect expression levels of inflammatory factors (*IL-1b, IL-6,* and *TNF-a*) and antioxidant stress factors (superoxide dismutase [SOD] and heme oxygenase-1 [HO-1]). The reagent kits and primers used in RT-qPCR are listed in Supplementary Material. The remaining tissue was fixed in neutral-buffered formalin, embedded in paraffin, and subjected to immunohistochemical analysis for Keap1 and Nrf2 expression. ImageJ software was used to select the yellow-stained immunohistochemical reaction areas on the images, followed by measurement of the mean optical density and semi-quantitative analysis of these regions.

### 2.5 Data analysis

Data are presented as mean ± SD. Data were analyzed using GraphPad Prism 9 software (GraphPad Software Inc., San Diego, CA, United States). One-way ANOVA and Student's t-test were used to determine the statistical significance between groups and a p-value less than 0.05 was considered statistically significant.

## 3 Results

### 3.1 Preparation and characterization of DL film

#### 3.1.1 FT-IR analysis of Gel-DOPA

**FIGURE 2:**
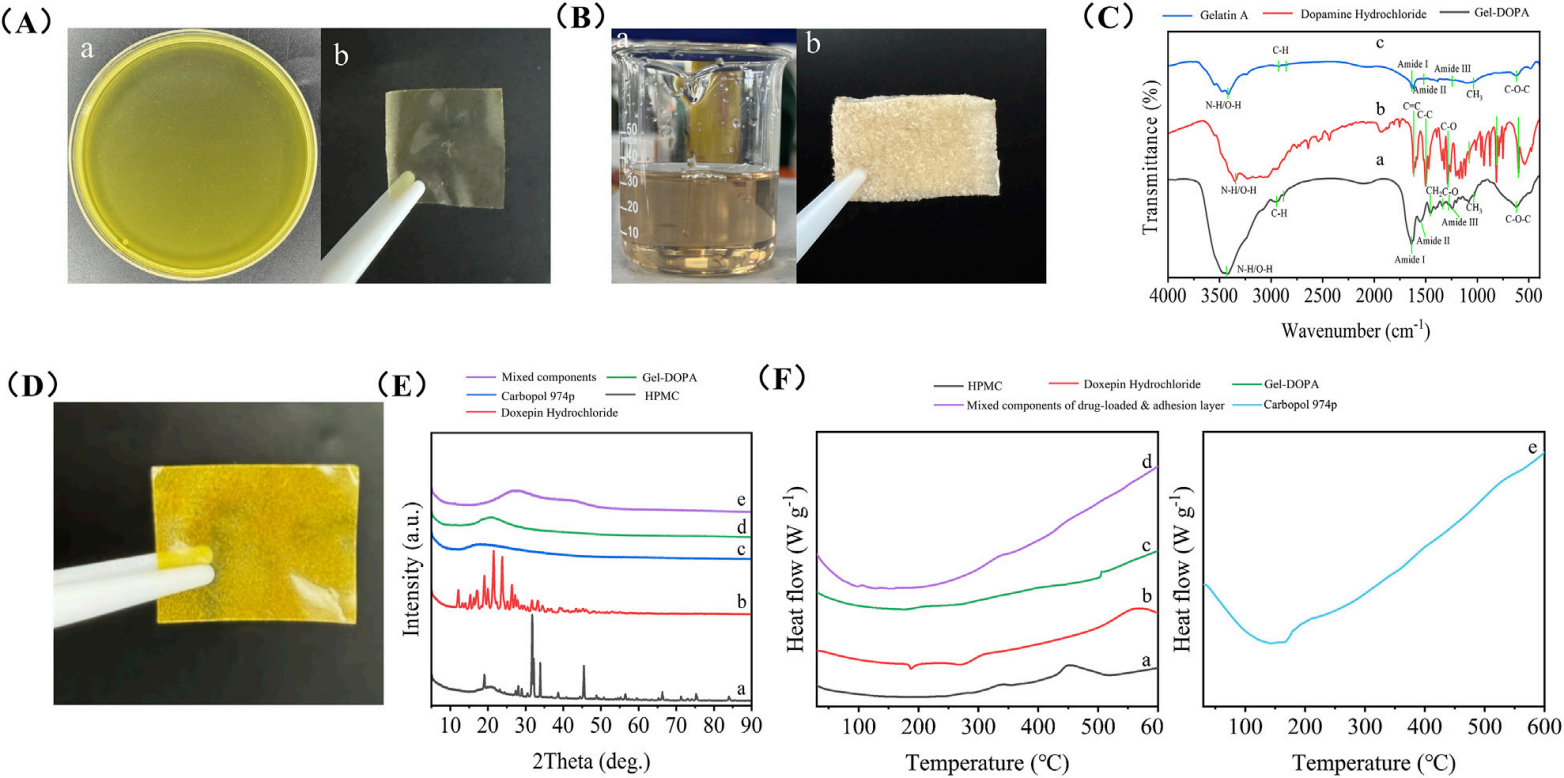
Preparation and characterization of a wet adhesive double layer oral film loaded with doxepin hydrochloride. **(A)** Solution of the backing layer (a) and its morphology after drying (b). **(B)** Gel-DOPA solution (a) and its morphology after freeze-drying (b). **(C)** FTIR spectra of Gel-DOPA (a), dopamine hydrochloride (b) and Gelatin A. **(D)** Photo of the double layer oral film loaded with doxepin hydrochloride. **(E)** XRD patterns of HPMC (a), doxepin hydrochloride (b), Carbopol (c), Gel-DOPA (d) and mixed components of drug-loaded and adhesion layer (e). **(F)** DSC analysis of HPMC (a), doxepin hydrochloride (b), Gel-DOPA (c), mixed components of drug-loaded and adhesion layer (d) and Carbopol (e).

As shown in Figure 2C, the absorption band of Gel-DOPA near 3,434 cm^-1^ corresponds to N-H/O-H stretching vibrations. Gelatin inherently exhibits a broad band owing to protein hydrogen bonding interactions. The vibrations of the phenolic hydroxyl groups (-OH) and amines (-NH-) in polydopamine also fell within this range. The superposition of these two factors resulted in a strong and broad signal in this region. The band at 1,635 cm^-1^ (amide I, C=O and C-N stretching vibrations) serves as a sensitive indicator of protein secondary structures, such as β-sheets. The band at 1,550 cm^-1^ (amide II) arises from N-H bending vibrations and C-N stretching vibrations, with additional contributions from aromatic ring vibrations (e.g., C=C stretching in benzene rings) in polydopamine. The band at 1,240 cm^-1^ (amide III, C-N and N-H vibrations) provides critical information about protein conformations. These characteristic bands are attributed to Gel-DOPA. The emergence of a new C-O vibration peak indicates the successful grafting of catechol groups from dopamine hydrochloride onto the gelatin molecular chains. The band at 2,945 cm^-1^ represents asymmetric stretching vibrations of CH_2_, while the band at 2,875 cm^-1^ corresponds to symmetric stretching vibrations of CH_2_. Bending and rocking vibrations of CH_2_ are observed at 1,455 and 1,333 cm^-1^, respectively. Finally, the band at 1,032 cm^-1^ corresponds to CH_3_ amide groups and the C-O-C band appears at 620 cm^-1^. This analysis conclusively demonstrates the successful synthesis of Gel-DOPA.

#### 3.1.2 XRD analysis

The sharp diffraction peaks of HPMC and doxepin hydrochloride indicated their highly crystalline nature (Figure 2E). In contrast, a broad hump was observed between 20° and 30° for carbomer 974P, gelatin-polydopamine, and the drug-loaded layer mixture reflected amorphous characteristics. The random molecular chain network of the carbomer, which is a cross-linked polyacrylic acid, inhibits crystalline ordering. After polydopamine modification, the original collagen triple-helix structure of gelatin was disrupted, forming a disordered, physically cross-linked network. The amorphous state of the drug-loaded mixture stems from complex intermolecular interactions; hydroxyl groups from HPMC, carboxyl groups from carbomers, and drug molecules engage in hydrogen bonding and ionic interactions that prevent molecular alignment. This amorphous structure enhanced the dissolution rate and facilitated drug release.

#### 3.1.3 DSC

DSC curves (Figure 2F) revealed distinct thermal behaviors of HPMC, doxepin hydrochloride, carbomer 974P, gelatin-polydopamine, and synthetic gels. The endothermic peak of carbomer 974P (e) near 160 °C was likely associated with the rupture of intramolecular hydrogen bonds. The dual endothermic peaks of doxepin hydrochloride (b) at 190 °C and 270 °C might correspond to its melting process and hydrochloride salt decomposition, respectively, while the exothermic bulge at 560 °C could represent the exothermic response from oxidative decomposition of organic carbon chains. The exothermic peak of HPMC (a) at approximately 450 °C was related to thermal oxidative decomposition of its cellulose backbone, with heat release from cleavage of methyl and hydroxypropyl substituents at elevated temperatures. The sharp exothermic peak of gelatin-polydopamine (d) at 505 °C might originate from carbonization exotherm of the polydopamine cross-linked network, where energy release occurs through aromatic ring structural reorganization above 500 °C. These results are also consistent with previous reports. (Khan et al., 2022; Kanwar et al., 2016; Ghaderi et al., 2019). The mixed component system (e) showed no distinct thermal events, indicating the formation of a stable amorphous composite system through intermolecular interactions (e.g., hydrogen bonding between HPMC hydroxyl groups and carbomer carboxyl groups, π-π stacking between gelatin-polydopamine and drugs), which suppressed characteristic thermal behaviors of individual components. This observation is consistent with the XRD results, suggesting that such an amorphous drug dispersion facilitates drug dissolution and enables uniform distribution within the film matrix.

#### 3.1.4 General characteristics of DL film

**FIGURE 3:**
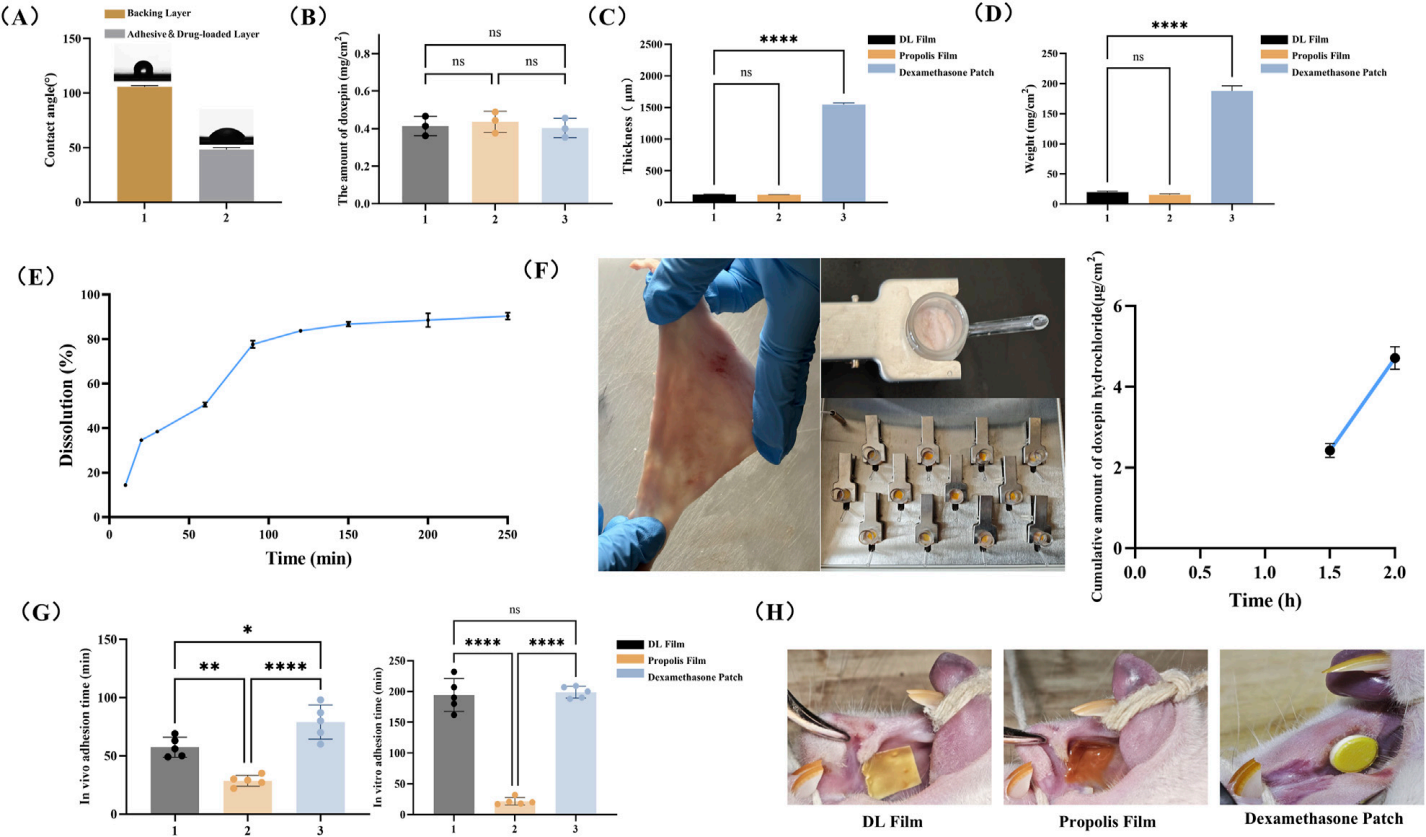
General Characteristics of the DL film. **(A)** WCA analysis of the DL film. **(B)** Detection of drug content in DL film. **(C)** Relative weight of DL Film, Propolis film and Dexamethasone patch. **(D)** Thickness measurement of DL film, Propolis film and Dexamethasone patch. **(E)** *In vitro* drug release profiles of DL film. **(F)** *Ex vivo* drug permeation profiles of DL film. Mucosal adhesion of three patches **(G)** and analysis of adhesion time *in vitro* and *in vivo* **(H)**. Data represent mean ± SD. *p < 0.05, **p < 0.01, ***p < 0.001.

WCA analysis confirmed that the backing layer is hydrophobic, whereas the drug-loaded and adhesive layers are hydrophilic. As shown in Figure 3A, the backing layer exhibited a significantly higher WCA (104.6° ± 3.2°, 1 s) compared with that of the drug-loaded layer (55.1° ± 0.7°, 1 s). The hydrophobicity of the backing layer helps prevent the dilution of the drug concentration caused by saliva washing in the oral cavity to a certain extent, thereby achieving a localized unidirectional release effect of the drug (Tehrani-Bagha, 2019). The hydrophilic surfaces of the drug-loaded and adhesive layers facilitate patch adhesion and drug release.

Furthermore, the drug content in DL membranes fabricated via 3D printing technology demonstrated strong homogeneity. As shown in Figure 3B, no significant differences were observed in the average drug content (0.4 mg/cm^2^) among three randomly selected batches of DL membranes.

The DL film developed in this study demonstrated an ultrathin profile of 128.3 ± 1.5 μm, comparable to propolis patches and significantly thinner than dexamethasone acetate patches (1,546.0 ± 26.9 μm) (Figure 3C). Moreover, its areal density was merely 19.8 ± 1.6 mg/cm^2^, substantially lower than that of dexamethasone patches (Figure 3D). These metrics highlight the notable thinness and lightweight characteristics of the film, which enhance patient comfort by minimizing foreign body sensations and improving mucosal conformity during localized administration. The results of the *in vitro* dissolution test demonstrated that doxepin hydrochloride was released gradually as the components of the drug-loaded layer dissolved, with nearly 80% of the drug released within 90 min (Figure 3E). Meanwhile, data from the *in vitro* permeability study indicated that the dissolved doxepin could permeate the local mucosal tissue. Although approximately 40% and 50% of the total doxepin hydrochloride was released at 0.5 h and 1 h, respectively, the amount permeating into the mucosa remained below the detection limit of the chromatographic assay (Figures 3E,F). However, at later time points, the drug levels within and beneath the mucosa showed a detectable increase (Figure 3F). This confirms that the drug is released gradually over time and can continuously permeate into the mucosal tissue.

An ideal buccal adhesive system requires adequate mucoadhesion to ensure localized retention and sustained therapeutic effects (Shipp et al., 2022). Therefore, we evaluated the *in vivo* and *in vitro* adhesion duration of the three patch types to assess the mucoadhesive performance of the DL film. As depicted in Figure 3G, the DL film incorporating the wet adhesive material Gel-DOPA demonstrated a significantly prolonged *in vivo* adhesion duration (average of 57 min) compared to that with propolis patches. *In vitro* adhesion testing showed comparable adhesion times between the DL film and the dexamethasone patch (194.2 min vs 198.8 min), both markedly exceeding that of propolis patches (21.6 min). This discrepancy likely stems from the absence of a backing layer in propolis patches, leading to rapid dissolution in high-moisture environments. The adhesion performance of three types of patches on rat buccal mucosa is shown in Figure 3H. It can be observed that although the dexamethasone patch demonstrates extended adhesion time, its lack of flexibility and fixed size prevent complete mucosal conformal adhesion and personalized dosing adaptation based on the specific dimensions of ulcers.

### 3.2 DL membrane promotes the healing of CIOM in rats

**FIGURE 4:**
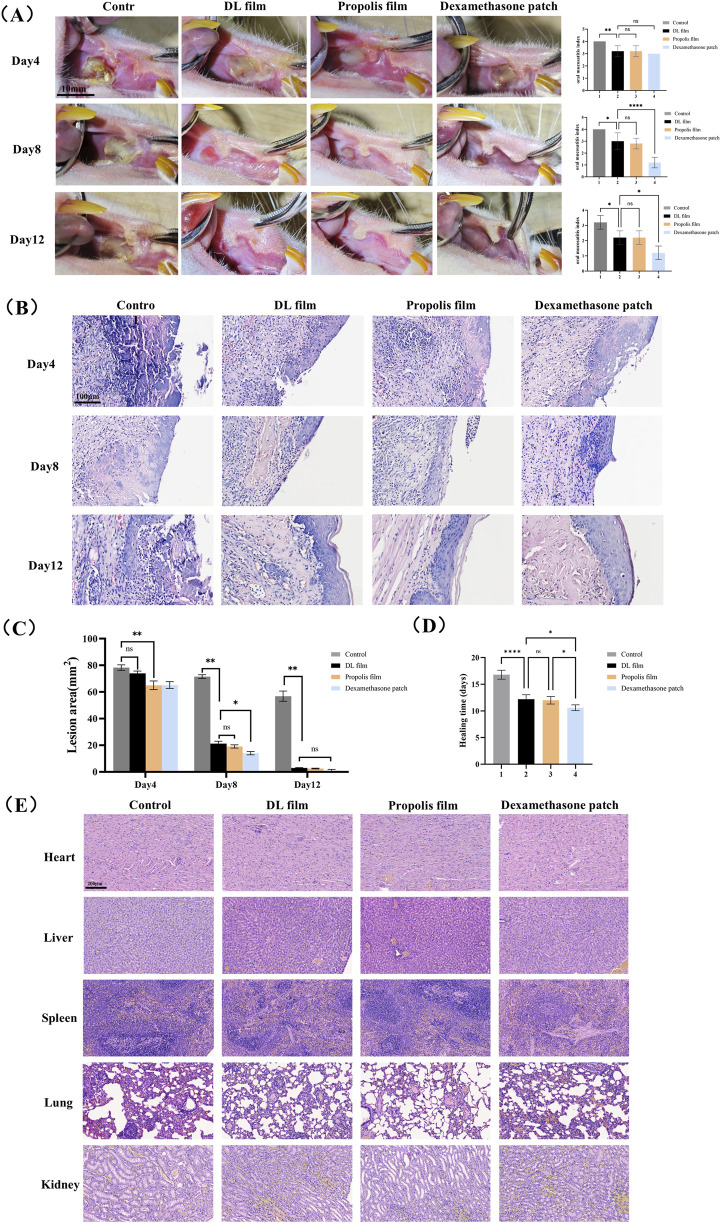
Efficacy and safety evaluation of DL film in the treatment of CIOM. **(A)** Gross observation photographs and mucositis scores during ulcer treatment in each group (n = 5, scale bar: 10 mm). **(B)** Area of ulcer lesion during treatment in each group. **(C)** The average healing time of lesion in each group. **(D)** H&E staining images of each group during lesion healing. **(E)** H&E staining images of major organs (heart, liver, spleen, lung, kidney) in each group after treatment. Data represent mean ± SD. *p < 0.05, **p < 0.01, ***p < 0.001.

As shown in Figure 4A, after 4 days of treatment, the inflammation scores in all treatment groups were significantly reduced, with macroscopic manifestations including markedly improved local redness and swelling at the ulcer site, formation of yellow pseudomembranes, and initial healing signs. The 5-FU group exhibited significant local congestion with abundant yellow inflammatory exudate and necrotic tissue in the defect area. After 8 days of treatment, the ulcer areas in all three treatment groups were significantly smaller than those in the 5-FU group. Although no significant difference in ulcer size was observed between the DL and propolis film groups, the dexamethasone patch group showed significantly smaller ulcer areas than those of the other three groups. The dexamethasone patch group demonstrated milder mucosal congestion and lower inflammation scores than those of the other groups. After 12 days of treatment, the ulcers in all three treatment groups were essentially healed, characterized by significantly reduced inflammatory infiltration in the subepithelial connective tissue, gradual coverage of the defect area by the neoepithelium, disappearance of pseudomembranes, and restoration of the epithelial architecture. In contrast, the untreated group displayed marked congestion, pseudomembranes, histopathological inflammatory cell infiltration, and disorganized epithelial layers (Figure 4B). Regarding healing time, the dexamethasone patch group showed the shortest average healing duration (10.6 days), while no significant difference was observed between the propolis and doxepin groups (12.0 days vs. 12.2 days) (Figures 4C,D). These results indicate that all three ulcer patches promoted ulcer healing, with dexamethasone patches demonstrating the strongest healing-promoting effect, whereas doxepin and propolis patches exhibited comparable therapeutic efficacy.

We also evaluated the tissue compatibility of the DL film with major organs (heart, liver, spleen, lungs, and kidneys) using H&E staining. H&E staining results demonstrated that after continuous administration until lesion healing, no significant tissue damage or pathological changes were observed in any major organ in the doxepin patch-treated group (Figure 4E). These findings indicate that the doxepin patch exhibits excellent biocompatibility and its topical application does not induce toxic effects in the organs.

### 3.3 DL film promotes the healing of CIOM by regulating oxidative stress and inflammatory responses

**FIGURE 5:**
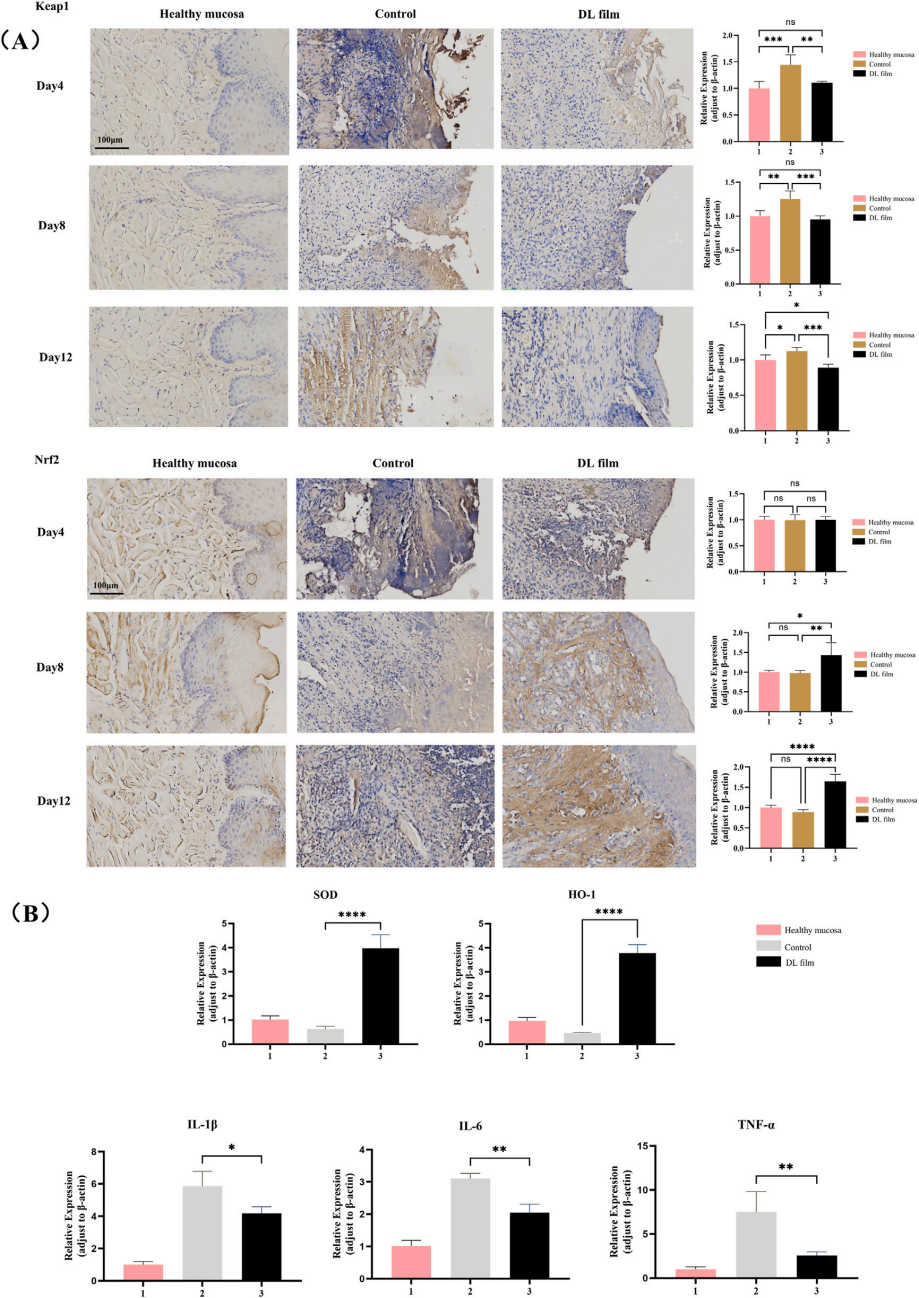
DL film promotes the healing of CIOM by regulating oxidative stress and inflammatory responses. **(A)** Immunohistochemical images and expression analysis of Nrf2 and Keap1 in CIOM lesion during DL film, propolis film treatment compared with control group. **(B)** SOD, HO-1, IL-1β, IL-6, TNF-α expression during DL film, propolis film and control treatment detected qRT-PCR. Data represent mean ± SD. *p < 0.05, **p < 0.01, ***p < 0.001.

We employed immunohistochemistry and RT-qPCR methods to detect the expression of related inflammatory factors and key components of the antioxidant system in lesioned tissues during DL patch treatment. Immunohistochemical findings further validated the regulatory effects of doxepin patches on the upstream Keap1/Nrf2 pathway, specifically by reducing Keap1 protein expression and thereby upregulating Nrf2 expression (Figure 5A). RT-qPCR results confirmed that treatment with doxepin patches upregulates the expression of intracellular antioxidant stress systems (SOD and HO-1), while reducing the expression of inflammatory cytokines (IL-1β, IL-6, and TNF-α) (Figure 5B).

## 4 Discussion

As one of the most prevalent complications of oncological treatment, CIOM can lead to pain, dysphagia, xerostomia, and dysgeusia. These symptoms substantially impair quality of life and may necessitate treatment interruption, thereby compromising therapeutic outcomes (Kusiak et al., 2020; Hong et al., 2019). Consequently, timely and effective management of patients with CIOM clinically important. Multiple clinical studies have confirmed that doxepin can achieve definitive analgesic effects and it is recommended as a medication for CIOM. However, no studies have reported the wound-healing effects of CIOM lesions, particularly established ulcerative lesions (Vélez et al., 2004). Currently, this drug is predominantly applied as a mouthwash, and there is a relative lack of suitable topical adhesive formulations for localized application. In this study, the combination of the drug doxepin with an adhesive system collectively demonstrated favorable effects in promoting ulcer healing, thereby expanding the application scope of this traditional antidepressant drug and providing new insights for the clinical management of CIOM.

Oral adhesive materials with high practical value are primarily oral mucosal patches or films. Their preparation methods include solvent casting, 3D printing, and electrospinning (Simões et al., 2021; Zhang et al., 2022). In additive manufacturing, 3D printing is a technology that fabricates solid components via layer-by-layer material accumulation based on 3D CAD data. It has been widely applied in the aerospace, architectural, and medical fields. The 3D-printed patches demonstrate unique advantages, such as customizable drug size/morphology and precise drug-release control. Since the U.S. Food and Drug Administration approved 3D printing for pharmaceutical applications, research and implementation of this technology have considerably expanded. Takashima et al. developed apigenin-loaded oral mucosal patches by 3D printing and confirmed their chemo-preventive efficacy against oral cancer (Takashima et al., 2022). Tatsuaki et al. successfully fabricated catechins-loaded patches for oral the treatment of 3D printing (Tagami et al., 2019). Given that the area and morphology of CIOM lesions often vary from person to person, we selected the patch formulation as it allows patients to make personalized adjustments based on their specific conditions and is easy to use. The use of 3D printing technology enabled the development of a locally administered formulation with stable drug content and ease of processing. Furthermore, the incorporation of a bioinspired wet-adhesive component significantly enhances the retention time of the drug in the oral cavity. The integration of these multiple advantages enables the drug to adhere strongly to the oral mucosal surface for precise drug delivery, thereby maximizing the therapeutic efficacy of the active ingredient, doxepin, and achieving favorable treatment outcomes.

**FIGURE 6:**
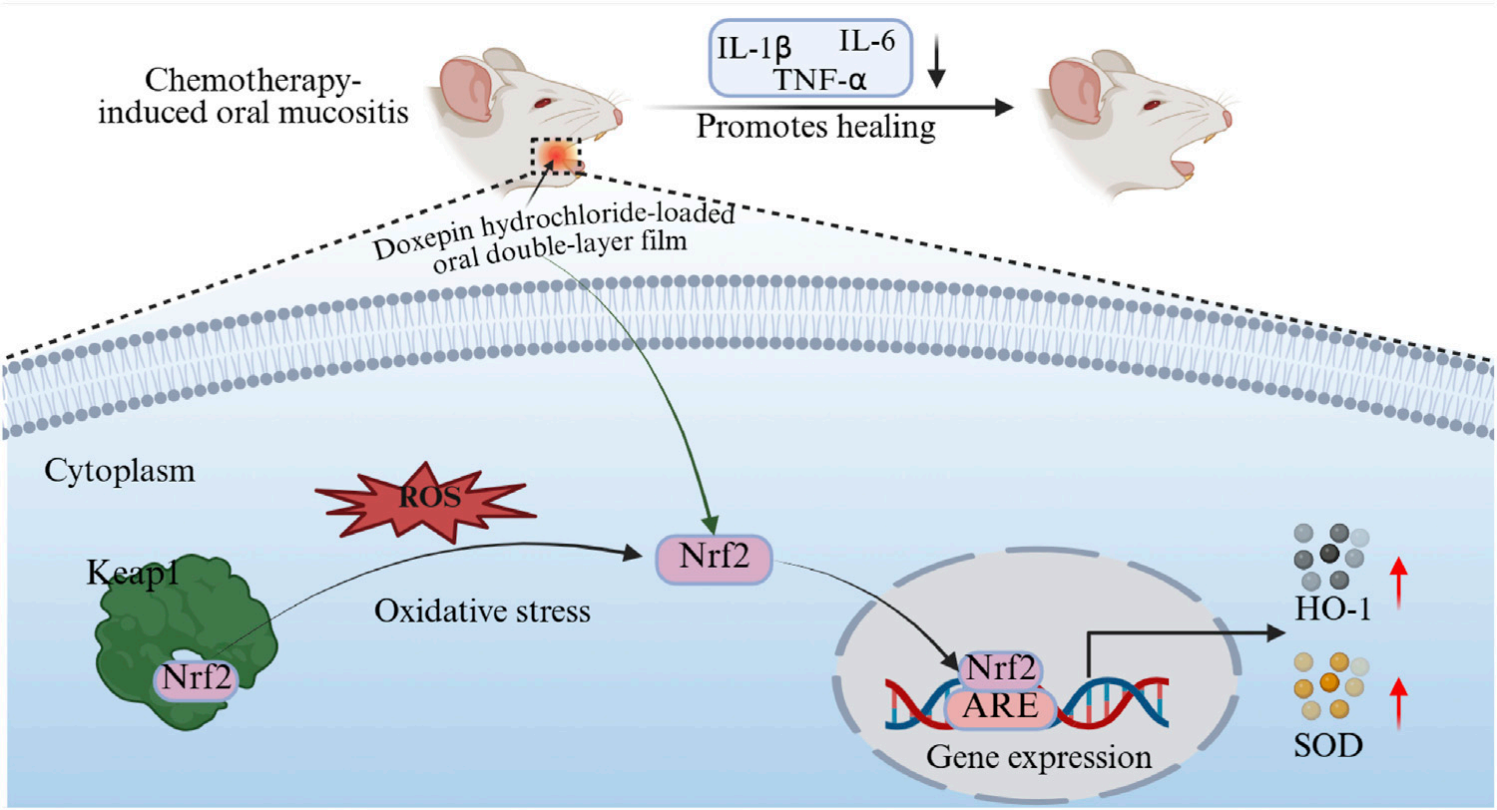
Schematic diagram of the DL membrane’s mechanism in promoting CIOM healing.

Oxidative stress plays an important role in CIOM pathogenesis. Chemotherapeutic agents generate reactive oxygen species (ROS) that target tumor cell DNA, leading to cellular damage and death (Campos et al., 2014; Cinausero et al., 2017). However, ROS also acts on the DNA of normal cells and exert cytotoxic effects. Multiple studies have confirmed that excessive ROS-induced oxidative stress in the local tissues of CIOM disrupts the redox balance in the body, while ROS further exacerbates local inflammatory responses. Researches by Bossi et al. demonstrated that elevated levels of inflammatory cytokines, such as IL-1β, IL-6, and TNF-α, in the serum and saliva of patients with CIOM are positively correlated with oral mucosal toxicity (Bossi et al., 2016; Huang et al., 2022). These cytokines enhance NF-κB activation through positive feedback, initiate the MAPK pathway and activate the JNK pathway, ultimately leading to apoptosis of submucosal and basal cells (Cinausero et al., 2017). Thus, an oxidative stress imbalance can be regarded as the initiating factor of CIOM, whereas excessive local inflammatory responses aggravate disease progression. Nrf2 is a critical transcription factor in the antioxidant stress response of the body (Jeong et al., 2006). It is regulated by Keap1, which serves as an inhibitory protein that binds to Nrf2 in the cytoplasm under normal conditions, thereby keeping it inactive (Adams et al., 2000). During oxidative stress, Keap1 undergoes conformational changes, dissociates from Nrf2, and releases Nrf2. The liberated Nrf2 translocates into the nucleus, where it activates the expression of downstream antioxidant enzymes, such as SOD and HO-1, thereby exerting protective effects (Kongpetch et al., 2012). In this study, we confirmed that the therapeutic efficacy of doxepin patches in CIOM lies in the modulation of the Keap1/Nrf2-HO-1/SOD signaling axis. The DL film promoted CIOM healing by alleviating excessive oxidative stress and secondary inflammatory responses (Figure 6).

The drug has demonstrated clear efficacy and safety in animal studies, with a relatively well-defined mechanism of action. It exhibits superior adhesive properties and customization potential compared to existing commercially available drugs, which contributes to its translational prospects. Additionally, most materials used in the drug preparation are FDA-approved substances applicable as food and pharmaceutical additives, further enhancing the likelihood of successful material translation in later stages. The production process and quality control are equally critical for drug translation. The formulation of medicated ink for 3D printing and the corresponding printing parameters demonstrated in this study are reproducible. The final shape of the printed patch can be customized as needed simply by adjusting the CAD file of the 3D printer. Furthermore, in large-scale commercial production, the introduction of multi-nozzle printing systems can enhance production efficiency. Therefore, we believe this study possesses strong commercial scalability. However, this study has not yet conducted clinical trials on this doxepin patch to observe its clinical efficacy, which represents a limitation of the current research. Additionally, while no significant adverse effects of the drug were observed in this study, this aspect also requires further observation in clinical trials. Given the large number of patients suffering from chemotherapy-induced oral mucositis in clinical practice, we believe this drug holds significant promise for clinical translation. Future work will involve clinical trials and further translation of this drug.

## 5 Conclusion

In conclusion, we synthesized a double-layer oral film loaded with doxepin hydrochloride using 3D printing technology to treat CIOM. The 3D printing enabled uniform drug distribution within the patch while maintaining a thin and flexible structure. The incorporation of Gel-DOPA as a wet-adhesive material ensured a strong mucosal adhesion to the oral cavity. Zein, HPMC, and carbomer in the raw materials are all excellent biocompatible materials. When properly formulated, the pharmaceutical preparation showed no significant toxicity. By featuring a specialized backing layer, the film achieved unidirectional sustained drug release. In rat CIOM models, the film demonstrated therapeutic efficacy by promoting lesion healing through antioxidant and anti-inflammatory mechanisms. Given these advantages, DL films are promising for clinical applications as therapeutic agents for CIOM and holds promising translational prospects.

## Data Availability

The original contributions presented in the study are included in the article/Supplementary Material, further inquiries can be directed to the corresponding authors.
