# Supplementary material for: A double layer oral film loaded with doxepin hydrochloride for the treatment of chemotherapy-induced oral mucositis
Source: Front Pharmacol. 2025 Oct 17;16:1673190. doi: 10.3389/fphar.2025.1673190 (PMC12575273; doi:10.3389/fphar.2025.1673190)
Supplement: Supplementary file 1 [file Table1.docx]

Total RNA was isolated using RNA Easy Fast Tissue/Cell Kit (TIAN BIOTECH BEIJING Co., Ltd) in accordance with manufacturer’s instructions. RNA was reverse transcribed into cDNA using the PreScript Ⅲ RT ProMix For qPCR (EnzyValley Biotechnology Co., Ltd). After mixing template/primers with 2×Robust SYBR Green qPCR Promix, quantitative real-time PCR was performed using a. The mRNA expression level of the target gene was normalized to the housekeeping gene β-actin. Relative expression of a gene was calculated by the 2^-ΔΔCt^ method. Primers for qRT-PCR were provided by Sangon Biotech (China) and were listed in Table 1.

| Primers |  | Sequence (5′→3′) |
| --- | --- | --- |
| *IL-1β* | FORWARD | CTGTGACTCGTGGGATGATG |
|  | REVERSE | GGGATTTTGTCGTTGCTTGT |
| *IL-6* | FORWARD | CCCCAACTTCCAATGCTCTCC |
|  | REVERSE | AGCACACTAGGTTTGCCGAG |
| *TNF-α* | FORWARD | AAACTCGAGTGACAAGCCCGTA |
|  | REVERSE | AAATGGCAAATCGGCTGACGGT |
| HO-1 | FORWARD | CACGCATATACCCGCTACCT |
|  | REVERSE | AAGGCGGTCTTAGCCTCTTC |
| SOD | FORWARD | AGATGACTTGGGCAAAGGTG |
|  | REVERSE | CAATCCCAATCACACCACAA |
| *β-actin* | FORWARD | TGCTATGTTGCCCTAGACTTCG |
|  | REVERSE | GTTGGCATAGAGGTCTTTACGG |

**Table1.** Sequence of primers used for qRT-PCR.
